# The Glucagon-Like Peptide 1 Analogue Exendin-4 Attenuates the Nicotine-Induced Locomotor Stimulation, Accumbal Dopamine Release, Conditioned Place Preference as well as the Expression of Locomotor Sensitization in Mice

**DOI:** 10.1371/journal.pone.0077284

**Published:** 2013-10-18

**Authors:** Emil Egecioglu, Jörgen A. Engel, Elisabet Jerlhag

**Affiliations:** Institute of Neuroscience and Physiology, Department of Pharmacology, The Sahlgrenska Academy at the University of Gothenburg, Gothenburg, Sweden; University of Bordeaux, France

## Abstract

The gastrointestinal peptide glucagon-like peptide 1 (GLP-1) is known to regulate consummatory behavior and is released in response to nutrient ingestion. Analogues of this peptide recently emerged as novel pharmacotherapies for treatment of type II diabetes since they reduce gastric emptying, glucagon secretion as well as enhance glucose-dependent insulin secretion. The findings that GLP-1 targets reward related areas including mesolimbic dopamine areas indicate that the physiological role of GLP-1 extends beyond food intake and glucose homeostasis control to include reward regulation. The present series of experiments was therefore designed to investigate the effects of the GLP-1 receptor agonist, Exendin-4 (Ex4), on established nicotine-induced effects on the mesolimbic dopamine system in mice. Specifically, we show that treatment with Ex4, at a dose with no effect *per se*, attenuate nicotine-induced locomotor stimulation, accumbal dopamine release as well as the expression of conditioned place preference in mice. In accordance, Ex4 also blocks nicotine-induced expression of locomotor sensitization in mice. Given that development of nicotine addiction largely depends on the effects of nicotine on the mesolimbic dopamine system these findings indicate that the GLP-1 receptor may be a potential target for the development of novel treatment strategies for nicotine cessations in humans.

## Introduction

Nicotine addiction, a major health problem, cause a wide range of serious effects such as cancer, chronic obstructive pulmonary disease and cardiovascular disease [1]. Development of this chronic and relapsing disease largely depends on the effects of nicotine on the mesolimbic dopamine system (for review see [2-4]). By elucidating the complex neurobiological mechanisms involved in the nicotine-induced activation of the mesolimbic dopamine system, such as locomotor stimulation, accumbal dopamine release, expression of conditioned place preference (CPP) and expression of locomotor sensitization, new targets for treatment of nicotine abuse and smoking cessation can be identified. Interestingly, a novel role for gut-brain signals known to control hunger and satiation has recently emerged, namely in the regulation of reward induced by addictive drugs such as nicotine [5]. 

The gut-brain hormone glucagon-like peptide 1 is secreted in response to nutrient ingestion [6] from enteroendocrine L-cells of the intestinal mucosa [7]. It is also produced in the central nervous system, specifically in neurons in the nucleus tractus solitarius (NTS) that project throughout the brain to areas such as the hypothalamus and mesolimbic areas [8-11]. Glucagon-like peptide 1 is known to regulate food intake as well as body weight [12-14] via GLP-1 receptors expressed in the hypothalamus [15] and the NTS [16]. In addition to the hypothalamus and NTS, GLP-1 receptors are expressed in several brain areas such as the reward nodes ventral tegmental area and nucleus accumbens [8,9], implicating that GLP-1 may have a role in reward regulation. Interestingly, GLP-1 neurons in the NTS project directly to the ventral tegmental area as well as the core and shell of nucleus accumbens [17]. The findings showing that activation of GLP-1 receptors in these areas reduces the intake of highly-palatable foods in rodents [17], suggest that these receptors may be involved in stimulation of the mesolimbic dopamine system. Given that common mechanisms regulate food and drug reward [5] we hypothesize that the GLP-1 signaling system, in addition to homeostatic control of food intake and glucose control, also could be involved in nicotine-induced reward. Indeed, it was recently shown that the GLP-1 analogue, exendin-4 (Ex4), suppressed the alcohol-induced locomotor stimulation, accumbal dopamine release and CPP for alcohol as well as reduced alcohol consumption and alcohol seeking behavior in rodents [18]. Supportively, Ex4 attenuates the ability of amphetamine as well as cocaine to cause a locomotor stimulation, accumbal dopamine release and induce a CPP in mice [19]. The present series of experiments was designed to evaluate the effects of Ex4 on the well established parameters reflecting activation of the mesolimbic dopamine system, namely nicotine-induced locomotor stimulation, accumbal dopamine release and CPP in mice. Furthermore, the effects of Ex4 on nicotine-induced expression of locomotor sensitization were investigated. The results of the presented experiments herein may be of clinical interest especially in view of that FDA-approved GLP-1 analogues, such as exenatide and liraglutide, are approved for the treatment of diabetes type II and thus tentatively could be used as novel treatments of nicotine addiction.

## Materials and Methods

### Animals

Adult post-pubertal age-matched male NMRI mice (8-12 weeks old and 25-40 g body weight; Charles River, Sulzfeld, Germany) were used. All mice were group housed and maintained at a 12/12 hour light/dark cycle (lights on at seven am). Tap water and food (Normal chow; Harlan Teklad, Norfolk, England) were supplied *ad libitum*, except during the experimental setups. The study was carried out in strict accordance with the recommendations in the Swedish Animal Welfare Act and all experiments were approved by The Swedish Ethical Committee on Animal Research in Gothenburg. All efforts were made to minimize animal suffering, and to reduce the number of animals used. All animals were allowed to acclimatize at least one week before the start of the experiments.

### Drugs

Nicotine ditartrate (Sigma-Aldrich; Stockholm, Sweden) was dissolved in vehicle (0.9% sodium chloride solution), and sodium bicarbonate was added until the pH was natural. The selected dose of nicotine, 0.5 mg/kg, was based on a previous dose response study where this dose increased the locomotor activity in mice [20]. Nicotine was administered (IP) fifteen minutes prior to initiation of the experiment. Exendin-4 (Tocris Bioscience, Bristol, UK) is a peptide with the amino acid sequence HGEGTFTSDLSKQMEEEAVRLFIEWLKNGGPSS GAPPPS. Exendin-4 has previously been established as a GLP-1 receptor agonist [21] and distribution studies show that the CNS binding of Ex4 is identical to GLP-1 [22]. The Ex4 dose used in the alcohol related experiments conducted in mice was selected based on the results from previous locomotor experiments, where 2.4 μg/kg was the highest dose that did not affect locomotor activity *per se* and this dose blocked the rewarding properties of alcohol in mice [18]. The selected dose of Ex4 did not affect the gross behavior of the mice in any of the experiments preformed. Exendin-4 was dissolved in vehicle (0.9% sodium chloride) and stored in aliquots at -20 degrees C before use. The Ex4 was administered intra peritoneal (IP) 10 minutes prior to the start of each experiment. A balanced or within subject design was used for all drug challenges. 

### Locomotor activity experiments

Locomotor activity was recorded as described previously [23]. In brief, locomotor activity was registered in eight sound attenuated, ventilated and dim lit locomotor boxes (420 x 420 x 200 mm, Kungsbacka mät- och reglerteknik AB, Fjärås, Sweden). Five by five rows of photocell beams, at the floor level of the box, creating photocell detection allowed a computer-based system to register the activity of the mice. Locomotor activity was defined as the accumulated number of new photocell beams interrupted during a 60-minute period. 

The mice were allowed to habituate to the locomotor activity box one hour prior to drug challenge. The effects of Ex4 (2.4 μg/kg, IP) on nicotine-induced (0.5 mg/kg, IP) locomotor stimulation were investigated. This dose of Ex4 was previously determined as the highest dose with no effect *per se* [18]. Ex4 was administered ten minutes prior to nicotine and the activity registration started fifteen minutes thereafter. Each mouse received one treatment combination creating the following treatment groups vehicle-vehicle (Veh-Veh), Ex4-vehicle (Ex4/Veh), vehicle- nicotine (Veh-Nic) or Ex4-nicotine (Ex4-Nic) (n=8 per treatment combination). Each mouse was only subjected to one experimental trial. 

### In vivo microdialysis and dopamine release measurements

For measurements of extracellular dopamine levels, mice were implanted unilaterally with a microdialysis probe positioned in the nucleus accumbens. The surgery was conducted two days prior to measurements of dopamine and was performed as described in detail previously [23]. In brief, the mice were anesthetized with isofluran (Isofluran Baxter; Univentor 400 Anaesthesia Unit, Univentor Ldt., Zejtun, Malta), placed in a stereotaxic frame (David Kopf Instruments; Tujunga, CA, USA) and kept on a heating pad to prevent hypothermia. The skull bone was exposed and one hole for the probe and one for the anchoring screw were drilled. The probe was randomly alternated to either the left or right side of the brain. The coordinates of 1.5 mm anterior to the bregma, ±0.7 lateral to the midline and 4.7 mm below the surface of the brain surface was used for the nucleus accumbens [24]. The exposed tip of the dialysis membrane (20 000 kDa cut off with an o.d./i.d. of 310/220 μm, HOSPAL, Gambro, Lund, Sweden) of the probe was 1 mm. All probes were surgically implanted two days prior to the experiment. After surgery the mice were kept in individual cages (Macrolon III) until the day of actual microdialysis experiment.

The effect of systemic administration of Ex4 (2.4 μg/kg, IP) on nicotine-induced (0.5 mg/kg, IP) accumbal dopamine release was investigated using microdialysis in freely moving mice. On the day of the experiment the probe was connected to a microperfusion pump (U-864 Syringe Pump; AgnThós AB) and perfused with Ringer solution at a rate of 1.5 μl/minute. After one hour of habituation to the microdialysis set-up, perfusion samples were collected every 20 minutes. The baseline dopamine level was defined as the average of three consecutive samples before the first drug/vehicle challenge, and the increase in accumbal dopamine was calculated as the percent increase from baseline. After the baseline samples (-40 minutes until 0 minutes), mice were injected with Ex4 or vehicle (at 5 minutes), which was followed by a nicotine or vehicle injection (at 20 minutes). Following these drug administrations an additional eight 20 minute samples were collected. The following treatment groups (n=8 in each group) were tested: vehicle-vehicle (Veh-Veh), vehicle-nicotine (Veh-Nic), Ex4-vehicle (Ex4-Veh) and Ex4-nicotine (Ex4-Nic) (n=8 in each group).

The dopamine levels in the dialysates were determined by HPLC with electrochemical detection. A pump (Gyncotec P580A; Kovalent AB; V. Frölunda, Sweden), an ion exchange column (2.0 x 100 mm, Prodigy 3 μm SA; Skandinaviska GeneTec AB; Kungsbacka, Sweden) and a detector (Antec Decade; Antec Leyden; Zoeterwoude, The Netherlands) equipped with a VT-03 flow cell (Antec Leyden) were used. The mobile phase (pH 5.6), consisting of sulfonic acid 10 mM, citric acid 200 mM, sodium citrate 200 mM, 10% EDTA, 30% MeOH, was vacuum filtered using a 0.2 μm membrane filter (GH Polypro; PALL Gelman Laboratory; Lund, Sweden). The mobile phase was delivered at a flow rate of 0.2 ml/minute passing a degasser (Kovalent AB), and the analyte was oxidized at +0.4 V.

**Figure 1:**
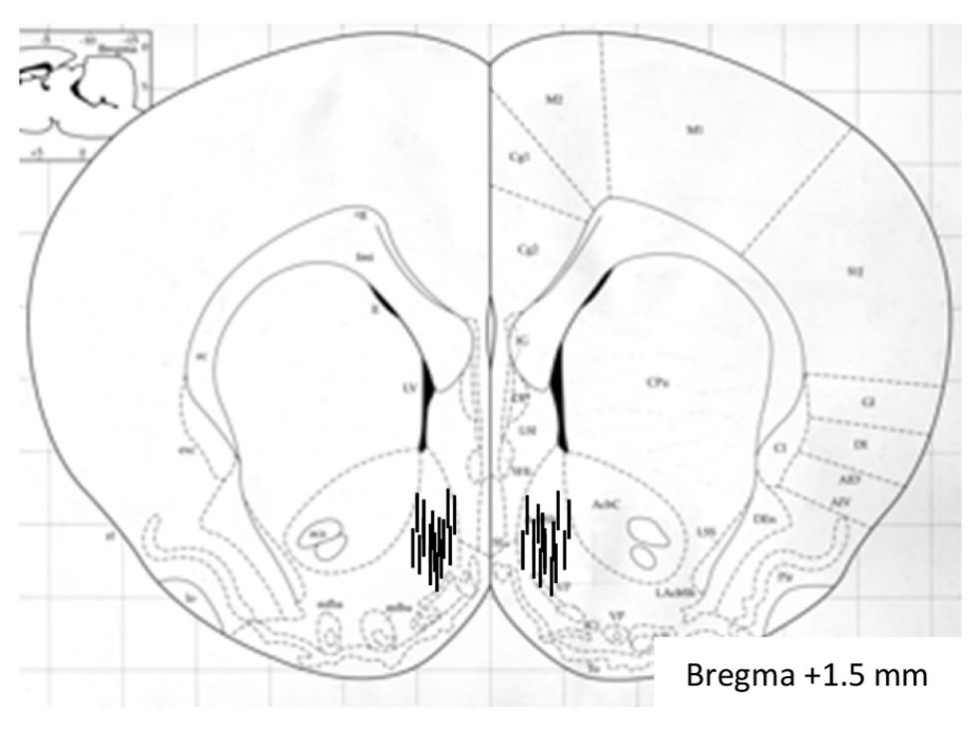
A coronal mouse brain section showing probe placements (illustrated by vertical lines) in the nucleus of mice used in the present study. The number given indicates millimeters anterior (+) from bregma.

After the microdialysis experiments were completed, the mice were decapitated, and probes were perfused with pontamine sky blue 6BX to facilitate probe localization. The brains were mounted on a vibroslice device (752M Vibroslice; Campden Instruments Ltd., Loughborough, UK) and cut in 50 μm sections. The location of the probe was determined by gross observation using light microscopy. The exact position of the probe was verified [24] and only mice with correct placements were used in the statistical analysis (Figure 1).

### Conditioned place preference

To evaluate the effects of Ex4 on the rewarding effects of nicotine, CPP tests were performed in mice as previously described [20,25]. In brief, a two-chambered CPP apparatus, with 45 lux illumination and distinct visual and tactile cues was used. One compartment was defined by black and white striped walls and by a dark laminated floor whereas the other had a white painted wooden floor and walls of wooden texture. The procedure consisted of pre-conditioning (day 1), conditioning (days 2-5), and post-conditioning (day 6). At preconditioning, mice were injected IP with vehicle and was placed in the chamber with free access to both compartments during 20 minutes to determine the initial place preference. Conditioning (20 minutes per session) was done using a biased procedure in which nicotine (0.5 mg/kg) was paired with the least preferred compartment and vehicle with the preferred compartment. All mice received one nicotine and one vehicle injection every day and the injections were altered between morning and afternoon in a balances design. At post-conditioning, mice were injected with Ex4 (2.4 μg/kg, IP) or an equal volume of vehicle solution and 10 minutes later placed on the midline between the two compartments with free access to both compartments for 20 minutes. The following two treatment groups were tested; nicotine-Ex4 (Ex4) and nicotine-vehicle (Veh) (n=8 in each group). Previous control experiments have shown that neither repeated vehicle administration nor the selected dose of Ex4 induces a CPP *per se* [18]. 

Conditioned place preference was calculated as the difference in % of total time spent in the drug-paired (i.e. least preferred) compartment during the post-conditioning and the pre-conditioning session. The present experiment was designed to reflect CPP expression rather than acquisition of CPP. Interestingly, it has been suggested that pharmacological agents that are administered only on the test day, as in the present design, should be considered as being potential candidates in the treatment of human drug craving (for review see [26]).

### Locomotor sensitization

Repeated administration of addictive drugs causes an increased locomotor response [27], i.e. a locomotor sensitization. The present sensitization experiment was designed in a similar way as reported by others [28] and investigates expression of nicotine-induced locomotor sensitization. Interestingly, expression of locomotor sensitization reflects the neurochemical alterations underlying important parts of addiction such as craving and compulsive drug taking [29]. The effects of acute Ex4 (2.4 μg/kg, IP) or an equal volume of vehicle on nicotine-induced (0.5 mg/kg, i.p.) locomotor sensitization was investigated. In these experiments nicotine or an equal volume of vehicle was administered each day for five subsequent days. At each of these days, the mice were allowed to habituate in the locomotor activity box for 60 minutes. The drug was thereafter injected and this was followed by 60-minute access to the locomotor activity box. The locomotor activity (defined as the cumulated number of new photocell beams interrupted during a 60-minute period) was registered the first treatment day (1), but not during treatment days 2-5. 72 hours following the last injection of the sub-chronic nicotine treatment (i.e. day 5) Ex4 or vehicle was administered acutely and the activity of the mice was investigated in the locomotor activity boxes. The rational for Ex4 administration following sub-chronic nicotine administration was to avoid co-administration of Ex4 and nicotine since we in the initial experiments show that Ex4 blocks the ability of nicotine to cause a locomotor stimulation. The locomotor activity was registered in the boxes described above and the locomotor sensitization was defined as the accumulated number of new photocell beams interrupted during a 60-minute period. Each mouse received only one treatment combination creating the following treatment groups; vehicle-vehicle (Veh-Veh), nicotine-vehicle (Nic-Veh), vehicle-Ex4 (Veh-Ex4) or nicotine-Ex4 (Nic-Ex4) (n=8 per treatment combination). 

### Statistical analysis

Locomotor activity data were evaluated by a one-way ANOVA followed by Bonferroni post-hoc tests. The microdialysis experiments were evaluated by a two-way ANOVA followed by Bonferroni post-hoc test for comparisons between different treatments and specifically at given time points. The condition place preference data were evaluated by an unpaired t-test. The locomotor sensitization data were analyzed with a two-way ANOVA followed by Bonferroni post-hoc tests. Data are presented as mean ± SEM. A probability value of P<0.05 was considered as statistically significant.

## Results

### Effects of Ex4 on nicotine-induced locomotor stimulation, accumbal dopamine release and expression of conditioned place preference in mice

**Figure 2:**
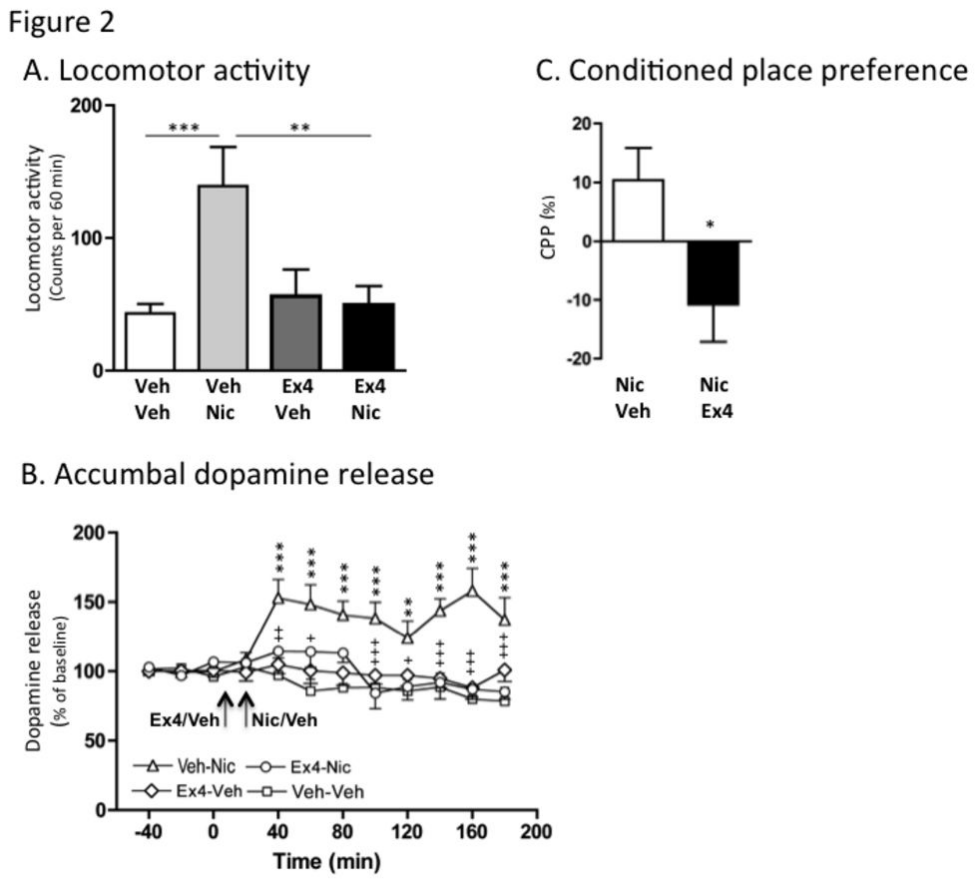
Exendin-4 attenuates nicotine-induced locomotor stimulation, accumbal dopamine release and conditioned place preference in mice. (A) Nicotine-induced (0.5 mg/kg IP) locomotor stimulation was attenuated by a single injection of Ex4 (2.4 μg/kg IP) (n=8 in each group; **P<0.01 and ***P<0.001, one-way ANOVA followed by a Bonferroni post-hoc test). (B) First we demonstrated a significant effect of nicotine (0.5 mg/kg IP) to increase dopamine release in comparison to vehicle treatment at time intervals 40-180 minutes (**P<0.01, ***P<0.01, veh-nic compared to veh-veh treatment). Secondly we showed that pre-treatment with Ex4 (2.4 μg/kg IP) attenuated the nicotine-induced increase in dopamine release compared to vehicle pre-treatment at time interval 40-60 and 100-180 minutes (+<0.05, ++P<0.01, +++P<0.01, Ex4-nic compared to veh-nic treatment). There was no difference in response between the veh-veh and Ex4-nic groups at a dose of Ex4 that had no effect *per* *se*. Arrows represent time points of injection of Ex4, vehicle and nicotine. Data analyzed with a Two-way ANOVA followed by a Bonferroni post-hoc test (n=8 in each group) (C) The nicotine-induced (0.5 m/kg IP) condition place preference (CPP) was attenuated by an acute single IP injection of Ex4 (2.4 μg/kg IP) in mice (n=8 in each group, *P<0.05, unpaired t-test). All values represent mean ± SEM. Arrow shows time for injections.

An overall main effect of treatment on locomotor activity was found in mice following systemic administration of nicotine (0.5 m g/kg) and Ex4 (2.4 μg/kg) (F(3,28)=8.11, *P*=0.0005; n=8 per group). As shown in Figure 2A, posthoc analysis revealed that nicotine significantly increased the locomotor activity compared to vehicle (*P*<0.001). This nicotine-induced locomotor activity was significantly reduced by pre-treatment with a single injection of Ex4 (P<0.01), at a dose that alone had no significant effect on locomotor activity compared to vehicle treatment (*P*>0.05). There was no difference in locomotor activity response in vehicle treated mice and Ex4-Nic treated mice (*P*>0.05).

Accumbal microdialysis measurements of dopamine in mice revealed an overall main effect of treatment (F(3,33)=61.15, P<0.0001) and treatment x time interaction (F(11,252)=3.45, P<0.0001), but not of time F(11,252)=1.28, P>0.05)(n=8 in each group). As shown in Figure 2B nicotine increased accumbal dopamine release relative to vehicle treatment at time interval 40-100 minutes (P<0.001), 120 minutes (P<0.01) and 140-180 minutes (P<0.001). This effect was attenuated by pre-treatment with Ex4 at time interval 40 minutes (P<0.01), 60 minutes (P<0.05), 100 minutes (P<0.001), 120 minutes (P<0.05) and 140-180 minutes (P<0.001). The selected dose had no significant effect on accumbal dopamine release compared to vehicle treatment at any time interval (*P*>0.05). There was no difference in accumbal dopamine response in vehicle treated mice and Ex4-Nic treated mice at any time interval (*P*>0.05). Only mice with correct probe placement in the nucleus accumbens shell were included in the statistical analysis (Figure 1). 

The nicotine-induced (0.5 mg/kg) (Nic-Veh) expression of CPP was significantly attenuated by an acute single injection of Ex4 (2.4 μg/kg) (Nic-Ex4) on the post-conditioning day compared to vehicle injection (*P*<0.05, n=8 in each group; Figure 2C).

### Effects of Ex4 on nicotine-induced expression of locomotor sensitization in mice

The locomotor sensitization data revealed an overall main effect of treatment (F(3,3)=10.57, *P*<0.0001), time F(1,42)=20.37, *P*=0.0005) as well as treatment x time interaction (F(1,42)=36.75, *P*=0.0008)(n=8 in each group). 

**Figure 3:**
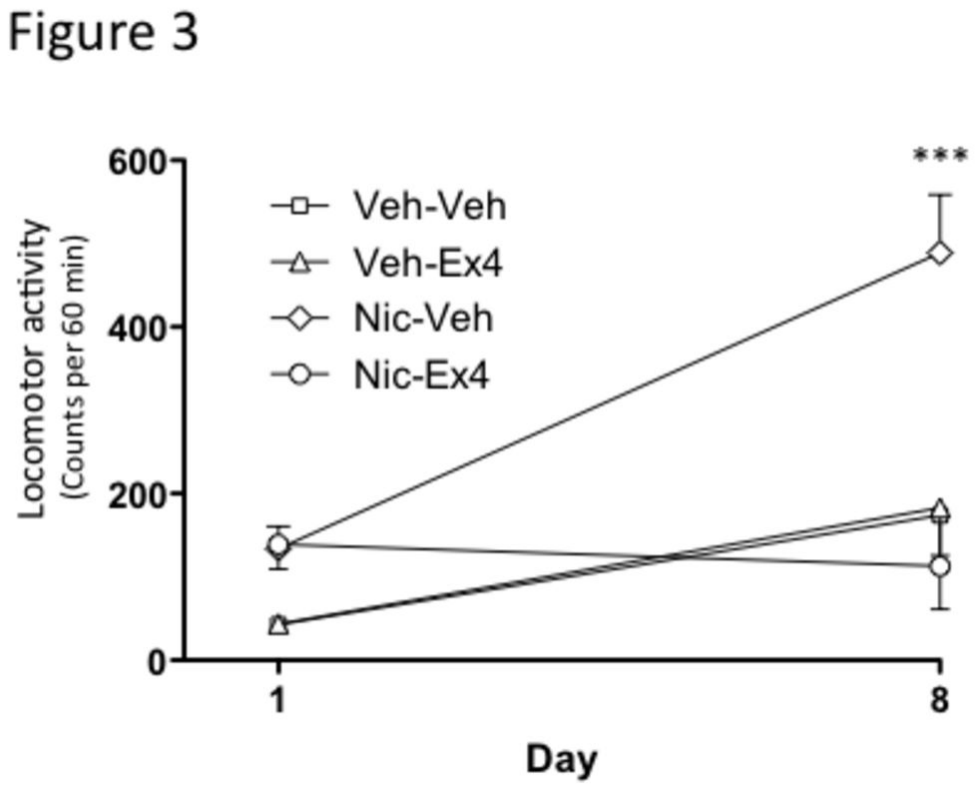
Exendin-4 blocks the nicotine-induced locomotor sensitization in mice. In the present experiment nicotine or vehicle for five days. 72 hours following this sub-chronic treatment Ex4 or vehicle was administered. Sub chronic nicotine treatment induced (0.5 mg/kg) a significant sensitization and this effect was attenuated by a single injection of Ex4 (2.4 μg/kg) (n=8; * ***P<0.001, two-way ANOVA followed by a Bonferroni post-hoc test). All values represent mean ± SEM.

As shown in Figure 3, posthoc analysis revealed that the locomotor activity is significantly higher in mice treated sub-chronically with nicotine (0.5 mg/kg) and acutely with vehicle (Nic-Veh) on the last day (i.e. day 8) as compared to vehicle (Veh-Veh) treated mice (*P*<0.001). Acute injection with Ex4 on the last day (i.e. day 8) blocked this nicotine-induced locomotor sensitization ((*P*>0.05) (Nic-Veh vs Nic-Ex4). The selected dose of Ex4 had no effect on locomotor activity per se (*P*>0.05) (Veh-Veh vs Veh-Ex4).. 

## Discussion

The present study provides novel evidence showing that GLP-1 receptors regulate nicotine-induced activation of the mesolimbic dopamine system in mice, suggesting that the physiological role of GLP-1 extends beyond control of glucose homeostasis and food intake [10,11]. Indeed, we demonstrate that the GLP-1 analogue, Ex4, attenuates the nicotine-induced locomotor stimulation, accumbal dopamine release and expression of CPP in mice. In accordance we show that Ex4 blocks the nicotine-induced expression of locomotor sensitization in mice, indicating that GLP-1 analogues deserve to be evaluated as a potential novel treatment target for smoking cessation and other nicotine addictions. 

Glucagon-like peptide 1 containing-fibers from the NTS target reward areas expressing GLP-1 receptors such as the VTA and N.Acc [8,9,17,30].. Given that development of nicotine addiction partly depends on the effects of nicotine on the mesolimbic dopamine system (for review see [2-4]) and that we here show that Ex4 suppresses established nicotine-induced effects on the mesolimbic dopamine system [26,27,31], we hypothesize that GLP-1 receptors within these reward nodes may mediate the rewarding properties of nicotine. Supportively, local administration of Ex4 into the VTA or N.Acc. reduces reward induced by and the intake of palatable foods [17,32] and accumbal administration of GLP-1 receptor antagonists cause hyperphagia, [33]. The data showing that local administration of Ex4 into the VTA reduces alcohol intake and alcohol-induced CPP in rodents [34], suggest that GLP-1 receptors within the VTA may be of importance for drug-induced reward. Glucagon-like peptide 1 receptors found in the hypothalamus and NTS have been shown to regulate GLP-1 dependent food intake [15,16] and glucose homeostasis [35], raising the possibility that GLP-1 receptors in these areas also could be of importance for nicotine-induced activation of the mesolimbic dopamine system and thus for reward. Albeit Ex4 crosses the blood-brain barrier [36] the possibility that the observed effects of Ex4 could be due to peripheral rather than central effects should be considered. In accordance, the ability of GLP-1 to reduce spontaneous meal size is dependent on vagal afferent signaling [37]. However, the exact circuits through which GLP-1 receptors regulate nicotine-reward needs to be further elucidated. Even though Ex4 has been suggested to affect other receptors than the GLP-1 receptor [38-40] the findings that a GLP-1 receptor antagonist blocks the ability of Ex4 to reduce the motivation to consume sucrose [32] supports our hypothesis that the reinforcing properties of rewards such as nicotine are mediated directly via GLP-1 receptors. The mechanisms through which GLP-1 receptors regulate the activity of mesolimbic dopamine neurons are to date unknown and needs to be investigated. 

Our present findings show for the first time an involvement of the GLP-1 receptor in nicotine-induced activation of the mesolimbic dopamine system as measured by locomotor stimulation, accumbal dopamine release and expression of CPP. In addition we showed that Ex4 blocks the nicotine-induced expression of locomotor sensitization in mice. Given that expression of locomotor sensitization has been suggested to reflect the neurochemical alterations underlying important parts of addiction such as craving and compulsive drug taking [29], GLP-1 receptors may have an important role in the development of addiction. To further investigate the role of GLP-1 receptors for nicotine addition the effect of Ex4 on nicotine self-administration should be investigated. In support of a role of GLP-1 receptors in addiction are the findings showing that Ex4 suppresses alcohol-induced locomotor stimulation, accumbal dopamine release as well as CPP in mice and reduces alcohol consumption and alcohol seeking behavior in rats [18], findings that have been corroborated by others [34]. Moreover, the findings that Ex4 attenuates amphetamine-induced locomotor stimulation, cocaine-induced CPP as well as psychostimulant-induced locomotor stimulation, accumbal dopamine release and CPP in rodents [19,41,42] are in line with the preset findings. Given that Ex4 also reduces the motivation to consume sucrose [32] it may be suggested that GLP-1 receptors have an important role for reward induced by addictive drugs as well as natural reward. Clinically available GLP-1 receptor analogues, such as exenatide and liraglutide, are approved for treatment of type II diabetes since they reduce gastric emptying, glucagon secretion as well as enhance glucose-dependent insulin secretion [43-46]. The present results indicate that these pharmaceutical agents deserve to be investigated as treatments of nicotine addiction, an entirely novel indication. 

Albeit previous studies have shown that Ex4, at a dose range similar to what was used in the present study, induces a condition taste aversion [47] the possibility that our results are due to aversion rather than reduced reward appears however less likely. Thus, repeated Ex4 treatment does not induce a conditioned place aversion in rodents [18,42], indicating that the Ex4 dose used is not aversive or induces nausea [48]. Moreover, Ex4 targets reward related parameters such as locomotor stimulation, accumbal dopamine release, CPP and locomotor sensitization not only for nicotine as shown in the present study, but also for alcohol, amphetamine and cocaine as shown elsewhere [18,19,41,42].

The present data support the hypothesis that common mechanisms, such as gut-brain hormones, regulate both food- and drug-induced activation of the mesolimbic dopamine system [5]. Previous studies show that the hunger hormones ghrelin and galanin are required for reward induced by nicotine [20,49]. Moreover, the plasma level of the anorexigenic peptide leptin is associated with nicotine craving in humans and a cholecystokinin antagonist reduces nicotine withdrawal in mice [50,51]. This raises an important question regarding the physiological role of gut–brain signals and metabolic regulators and their ability to influence not only food intake but also having a broader role in modulating reward related processes. In conclusion, our present results showing that Ex4 attenuates nicotine-induced locomotor stimulation, accumbal dopamine release, expression of CPP as well as expression of locomotor sensitization in mice, indicate that GLP-1 receptors may provide a unique target for the development of new treatments for smoking. 
